# Financial stress, health and malnourishment among older adults in India

**DOI:** 10.1186/s12877-023-04532-7

**Published:** 2023-12-15

**Authors:** Kajori Banerjee, Harihar Sahoo, Dipti Govil

**Affiliations:** 1 Department of Quantitative Techniques, Anil Surendra Modi School of Commerce (ASMSOC), SVKM’s Narsee Monjee Institute of Management Studies (NMIMS) Deemed-to-University, Mumbai, Maharashtra 400056 India; 2 Department of Family & Generations, International Institute for Population Sciences (IIPS), Mumbai, Maharashtra 400088 India

**Keywords:** Chronic morbidity, Financial stress, India, Malnourishment, Mental health

## Abstract

As India’s elderly population grows rapidly, there is a demand for robust policy tools for geriatric health management. This study focuses on unveiling the impact of financial stress and insecurity in diverse economic sectors on adult malnutrition in India. Further, we explore the connections of adult malnourishment with mental and physical health outcomes. Analysis has been done using data of 59,764 respondents aged 45 years and above from the Longitudinal Ageing Study in India (LASI- Wave I) (2017-19). A modified Malnutrition Universal Screening Tool (MUST) has been used to assess the risk of adult malnutrition. It categorizes malnutrition into Low Risk, High Risk Group 1 (HRG1-undernourished), and High Risk Group 2 (HRG2-over-nourished). Approximately 26% of adults were classified in HRG1, characterized by low body mass index, recent hospital admissions, and affiliation with food-insecure households. Around 25% adults belonged to HRG2 characterized by high body mass index and waist-to-hip ratio. The relative risk ratios from the multinomial logit generalized structural equation model indicate that the risk of being in HRG1 was 20–40% higher among respondents not presently working or receiving pension benefits, as well as those involved in agricultural work. The risk of being in HRG2 doubles if the respondent was diagnosed with some chronic disease during the last 12 months. A higher composite cognition score reduces the risk of being in HRG1 by 4%, while it increases the risk of being in HRG2 by 3%. Additionally, experiencing episodic depression raises the risk of being in HRG1 by 10%. Financial insecurity, particularly in the informal and agricultural sectors, coupled with poor mental health, hinders positive nutritional outcomes. Extending universal pro-poor policies to fortify food security in resource poor households and integrating mental health variables in nutrition policies can be beneficial to address adult malnourishment in India.

**Supplementary Information:**

The online version contains supplementary material available at 10.1186/s12877-023-04532-7.

## Introduction

Worldwide 1.9 billion adults are overweight and 462 million are underweight [1]. Adult malnutrition is often a consequence of adverse climatic conditions, childhood diseases, inadequate dietary and nutrient intake, household food insecurity, poor living conditions, and low financial capabilities [2–8]. The macro-economic consequences of malnourishment are serious and long-term from developmental, social, economic, and health care perspectives. To address this burden United Nations General Assembly adopted the UN Decade of Action on Nutrition from 2016 to 2025 in April 2016 which promotes policy commitments towards measurable action to combat all forms of malnutrition. In the Decade, the focus is on healthier and sustainable diets to eradicate all forms of malnutrition worldwide. Nutrition also forms one of the major pillars of a healthy life and is the second goal under the Sustainable Development Goals (SDG). However, physical health-related targets and goals often do not capture the effects of mental health. The deleterious effects of poor mental health and stress on malnutrition have been discussed in international forums and urgently require statistical introspection [9]. In recent times, there is a growing body of literature that highlights the linkage between the mental and physical health of individuals. This has become even more apparent during the ongoing global pandemic, as numerous individuals are experiencing heightened levels of stress and mental health challenges due to the physical and economic threats associated with COVID-19 [10]. Like all physical diseases, mental health issues are also closely associated with demography. Mental health issues are found to be prevalent among the older population and are often associated with physical co-morbidities and malnourishment [11, 12]. This makes an imperative domain of research to aid in policy formulation and build age-related preparedness in a country like India which has a rapidly growing elderly population.

Almost 1 in every 5 Indians is a senior citizen aged 60 years or older. The issue of ageing in India has grabbed global attention not only due to economic considerations but also needs of health and infrastructure planning and revisions. The draft National Policy for Senior Citizens 2020 highlights the revision of the pension programmes to enhance financial security in old age. The older working-age population (45–59 years) and nearing retirement or retired population (60 years and above) are not only subjected to drastic changes in physiological, pathological, social, and psychological conditions but also work to retirement transition. Those at the edge of work to retirement transition are often found to suffer from mental stress due to an array of issues such as financial insecurities, loneliness, changes in living arrangement, the incidence of physical ailments. Psychosomatic disorders born due to persistent stress, poor cognitive and mental health can often result in irregularity in nutrient and dietary intake and may result in severe malnutrition among adults [13]. However, studies investigating the linkages between malnourishment and mental health issues among adults in the work-retirement transitory phase are limited. Some studies emphasize the relationship between stress, psychological health, and eating behavior among adolescents who are considered to be at a higher risk of stress due to physical and psychological changes [14, 15]. However, another period of transition in life also comes during ageing when a person is at risk of coping with an array of physical and psychological transformations. The drastic transformation of nearing one’s economically active life and entering one’s retired life is often closely linked to various mental stressors. Understanding the impact of mental health at this age on nutritional outcomes is pertinent to developing impactful coping strategies and inputs to build age-friendly policies.

This paper has two main objectives. First, to assess the direct impact of physical and mental health status on the risk of adult malnourishment. Secondly, to elucidate the indirect effects of financial insecurity on the risk of adult malnourishment through degraded health conditions. The present paper has adopted an innovative approach to measure the risk of malnourishment among the adult population using a modified version of “Malnutrition Universal Screening Tool (MUST)” [16]. The tool includes physical manifestation of malnourishment through extreme body mass index, metabolic risk, food insecurity and acute illness that may result in loss of nutrition intake. These factors are considered to be direct predictors of malnourishment among adults [17]. In this research paper, we delineate adult malnutrition into three distinct categories: the Low Risk Group, High Risk Group 1, and High Risk Group 2. High Risk Group 1 encompasses the undernourished segment of the malnutrition continuum and is identified by individuals being underweight, admitted to the hospital in the past year and experiencing household food insecurity within the 12 months preceding the survey. On the other hand, High Risk Group 2 represents the over-nourished end of the malnutrition spectrum, characterized by individuals being overweight and having a high waist-to-hip ratio.

It is crucial to address both ends of the malnutrition spectrum, as numerous studies discuss the double burden of malnutrition in India [18]. The question at the heart of the study are: (i) “*What effects do chronic diseases and mental health conditions have on the two high-risk categories of adult malnutrition within the Indian population in the transitional age range between working life and retirement?”* and (ii)“*Does financial insecurities aggravate the risk of malnourishment among adults?”* The study hypothesizes that those suffering from chronic and mental health issues will suffer from some form of malnourishment irrespective of their financial well-being. The paper further hypothesizes that those who are currently engaged in the formal sector or are covered under some pension schemes providing financial safety will have better mental health and better nutritional status compared to their counterparts who are either retired or working in the informal sector. In absence of safety nets, especially for those working in the informal sector, many of the older citizens are predicted to suffer from poor mental health.

The study is timely as the adverse effects of psychosomatic and mental illness have aggravated during the COVID-19 pandemic. Several people have suffered the brunt of degraded mental health due to nationwide lockdowns, freeze in the real economy, social isolation, and related loneliness and threats of being infected by the virus. Financial insecurities have played a vital role in the degraded mental health condition of several adults during the ongoing pandemic. Elucidating how long-term mental illness can create issues in physical health in the form of malnutrition can assist in strengthening and revisiting existing public health policies. The findings from the paper is pertinent in managing and reforming adult health care programmes by highlighting the role of financial insecurities, physical and mental health in predisposing malnourishment.

### Data source

The study aims to understand the linkages of financial insecurities, health, and risks of adult malnourishment. This paper aims to understand this pathway for a population subjected to drastic changes in lifestyle, hence proposes to explore the population who are in their late work life or have entered their retired life. The First Wave of Longitudinal Ageing Study in India (LASI) (2017-19) provides data for 72,250 respondents out of whom 59,764 respondents aged 45 years and above with data on various malnutrition indicators are included in the current study. The survey covers adults who are at a mature stage of their work-life through retirement. The goal of studying adults at the work-retirement transitory phase can be achieved using the dataset. It is one of the most recent surveys that capture information related to work, mental health, psychiatric disorders of the pre and post-retirement age group. In the foreseeable future, the panel data collected by LASI can provide further insights on the issue of a causal relationship between financial insecurities, health, and adult malnutrition. Thus, the data is not only apt for the current paper but also if the paper is extended in the future, LASI can support robust statistical estimates to illustrate causal relationships by using cohort data.

### Outcome variable

We followed the established “Malnutrition Universal Screening Tool (MUST)” to evaluate the risk of malnutrition among the sampled adults from LASI. This tool was developed by the Malnutrition Action Group of the British Association for Parenteral and Enteral Nutrition (BAPEN) to effectively capture nutritional requirements and the risk of malnourishment among adults. It has been effective in predicting the risk of malnourishment and severe illness among adults [16, 19–21]. The “MUST” uses three indicators to measure the risk of malnourishment: body mass index (BMI), unplanned weight loss in the past 3–6 months, acute illness that may hamper nutritional intake for more than 5 days. This paper proposes some modifications in the screening tool to account for metabolic risks among adults. We have included four indicators to measure the risk of malnourishment among the adult population surveyed in LASI: Body Mass Index (BMI); Waist to hip ratio (WHR) to account for metabolic risk, food insecurity in the household (in last 12 months) and hospitalization (in last 12 months) as a proxy of acute illness that may hamper nutrition intake.

We recommend incorporating WHR as a malnutrition indicator, in addition to BMI, given its robust association with metabolic risk. Research indicates that, even with a normal BMI, an elevated WHR can lead to various obesity-related comorbidities, including cardiovascular diseases and chronic kidney diseases, highlighting its strong correlation with obesity and overweight [22, 23]. Food insecurity is measured using four components: had to reduce the size of meals or skip meals because there was not enough food at the household, was hungry but didn’t eat because there was not enough food at the household, did not eat for a whole day because there was not enough food at the household and lost weight in the last 12 months because there was not enough food at the household. These four components were collected with a time reference of 12 months. The food insecurity scale was constructed as per guidelines defined by FANTA [24]. In absence of a direct variable, food insecurity is taken as a proxy of unplanned weight loss. As per the MUST guidelines “history of decreased food intake” can be taken as a proxy variable for unplanned weight loss. Hospitalization in last 12 months is used as a proxy of “acute illness that may hamper nutritional intake”. Several studies have discussed about malnourishment in patients due to hospitalization [25, 26].

**Fig. 1:**
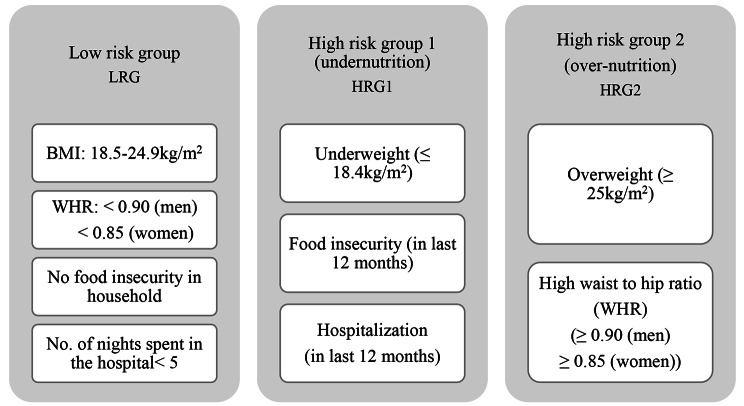
Construction of MUST categories

Instead of applying continuous scores like done in the “MUST”, we propose a three-category measurement method where categories under the low-risk group (LRG) are given a score of 0, high risk group 1 (HRG1) is given a score of 1 and high risk group 2 (HRG2) is given a score of 2. The rationale behind this modification is to address the double burden of adult malnutrition in India. The scourge of malnourishment in India is characterized by both over and under nutrition [18, 27]. Thus elucidating the risk factors that predispose both extremes of malnourishment is a policy priority. We have therefore separated these two types of malnourishment under the MUST indicator. Details of the three categories of the framework used for the present study is given in Fig. 1.

### Structural variables

Financial security is estimated using work status, mean income, retirement, and work-related pension coverage. Two main factors included to evaluate financial security are the industry the respondent is currently engaged in and availability and expectations about pension (for ever worked and currently working respondents).

Objective physical and mental chronic disease status is defined as those diagnosed with some chronic morbidity. These chronic morbidities may include: hypertension, diabetes, cancer/ malignant tumor, lung diseases like asthma chronic obstructive pulmonary disease/ chronic bronchitis, chronic heart diseases such as myocardial infarction, congestive heart failure, stroke, arthritis, osteoporosis, neurological/ psychiatric problems such as depression, Alzheimer’s/ Dementia, unipolar/ bipolar disorders, convulsions, Parkinson’s and high cholesterol. Episodic depression variable is added to the model to understand the impact of self-reported mental health status of respondents. The measure of episodic depression has been estimated using the questions asked in the well-established Composite International Diagnostic Interview Short Form (CIDI-SF) [28]. Composite cognition scale (CCS) is defined by respondent’s performance in various cognitive activities in various domains such as memory, verbal fluency, arithmetic, executive functioning, and object naming. Perceived life satisfaction index is controlled as a variable to assess overall quality of life and well-being of the respondents.

### Independent variables

Demographic and socio-economic variables that are controlled in the model are: gender (male/ female) age (45–59 years (pre-retirement age) and ≥ 60 years (post-retirement age)), place of residence, sex, marital status, living arrangement, religion, caste, education, household wealth status. These variables have been commonly employed as indirect predictors of malnutrition among adults, both on a global scale and within the context of India. [29–31]. The conceptual framework based on which the structural equation modelling is applied is provided in Figure A.1 (Appendix).

## Analytical strategy

The study uses the multinomial logit generalized structural equation modelling (ML-GSEM) to understand the causal relationship between adult malnutrition, financial insecurities in the work-retirement transitory phase, and physical and mental health. The rationale for using SEM is to disentangle the effects of financial insecurities, socio-economic characteristics, and mental health status. In an ordinary least square model or a logistic model, we cannot capture the endogeneity of the mental health variable. We assume that mental health is directly associated with financial security and socio-economic status. This relationship can be captured with ease in an ML-SEM framework.

### Salient findings

Out of the respondents aged 45 years and above 26.4% fell in high risk group 1 (HRG1) and 24.7% fell in high risk group 2 (HRG2). Table 1 provides a detailed distribution of the MUST categories by various characteristics. HRG1 had more males than females whereas HRG2 had more females than males. The percentage of respondents in HRG1 increased with age whereas for HRG2 it decreased with age. Around 33 to 40% of Scheduled caste/tribe members belonged to HRG1 whereas only 11–19% of them were in HRG2. Out of those who had higher level of education (secondary school and above) a higher percentage belonged to HRG2 compared to HRG1 whereas for those with lower levels of education, the trend was reversed. Those belonging to higher (richer) quintiles of MPCE had a higher percentage of members belonging to HRG2 and those belonging to lower (poorer) quintiles of MPCE had higher percentage of people belonging to HRG1.

**Table 1:** MUST indicators by various background characteristics for respondents aged 45 years and above, LASI, Wave 1, 2017-19

Characteristics	MUST			N (unweighted)
	LRG	HRG 1	HRG 2	
**Demographic characteristics**				
**Gender**				
Male	51.9	27.7	20.5	27,667
Female	46.3	25.3	28.3	32,097
**Age**				
45-49yrs	50.3	20.5	29.2	12,049
50-59yrs	49.5	20.9	29.6	19,148
60-69yrs	47.7	28.4	23.9	17,421
70yrs+	48.3	37.2	14.5	11,146
**Place of residence**				
Rural	50.6	31.7	17.7	39,116
Urban	44.8	14.1	41.1	20,648
**Religion**				
Hindu	49.4	26.7	23.9	43,795
Islam	46.4	25.4	28.3	7,104
Christian	47.7	29.7	22.5	6,037
Others	45.3	19.2	35.6	2,828
**Caste**				
Scheduled Caste	48.5	32.8	18.7	10,049
Scheduled Tribe	49.1	39.6	11.3	10,535
Other Backward Class	49.4	24.7	25.9	22,521
None of them	48.4	19.9	31.8	14,577
**Education**				
No/ Less than Primary	49.2	32.6	18.2	35,087
Primary Completed	48.9	21.6	29.5	7,951
Middle Completed	48.6	18.0	33.5	5,710
Secondary School/Matriculation	51.3	12.4	36.3	5,306
Higher Secondary/Intermediate	46.8	16.4	36.9	2,497
Higher education	44.3	6.3	49.4	3,212
**MPCE**				
Poorest	48.2	34.9	16.9	11,828
Poorer	46.6	33.0	20.4	12,058
Middle	51.7	24.2	24.2	12,064
Richer	50.3	21.2	28.5	12,043
Richest	47.5	16.6	36.0	11,771
**Living arrangements**				
Living alone	48.0	33.4	18.6	2,127
Living with spouse and/or others	50.0	26.3	23.7	9,137
Living with spouse and children	49.7	23.3	27.0	34,702
Living with children and others	46.8	31.0	22.2	11,328
Living with others only	43.7	40.2	16.1	2,470
**Financial security** **Work status**				
not currently working-never worked	44.6	21.7	33.7	16,380
ever worked but not currently working with pension	53.0	14.5	32.5	1,916
ever worked but not currently working without pension	45.4	34.4	20.2	13,841
working-agriculture	54.4	31.4	14.2	14,747
working-business etc.	48.4	17.1	34.5	5,359
working-salaried expecting pension	46.5	13.1	40.4	1,348
working-salaried not expecting pension	53.3	18.2	28.6	6,115
**Mental/ Physical health** **Diagnosed with chronic disease**				
No	52.3	30.0	17.7	31,941
Yes	44.9	22.2	32.9	27,823
**Cognition**				
Low	44.5	44.2	11.3	3,352
Below average	47.9	37.2	14.9	8,838
Average	50.0	24.1	25.9	26,837
Above average	49.5	13.2	37.3	14,467
**Episodic depression**				
No major symptoms	49.0	26.4	24.6	55,377
Major symptoms	46.8	26.2	27.0	4,387
**Life satisfaction Index**				
0	48.5	30.8	20.7	3,886
1	45.3	36.0	18.7	1,682
2	47.4	32.8	19.8	2,626
3	46.6	28.0	25.4	2,195
4	41.9	33.0	25.1	2,835
5	47.9	32.9	19.2	4,494
6	47.5	29.0	23.6	4,252
7	49.5	28.7	21.9	3,659
8	49.0	27.0	24.0	6,642
9	50.1	23.0	26.9	5,214
10	50.3	21.4	28.2	21,839
**Total**	**48.9**	**26.4**	**24.7**	**59,764**

**Fig. 2:**
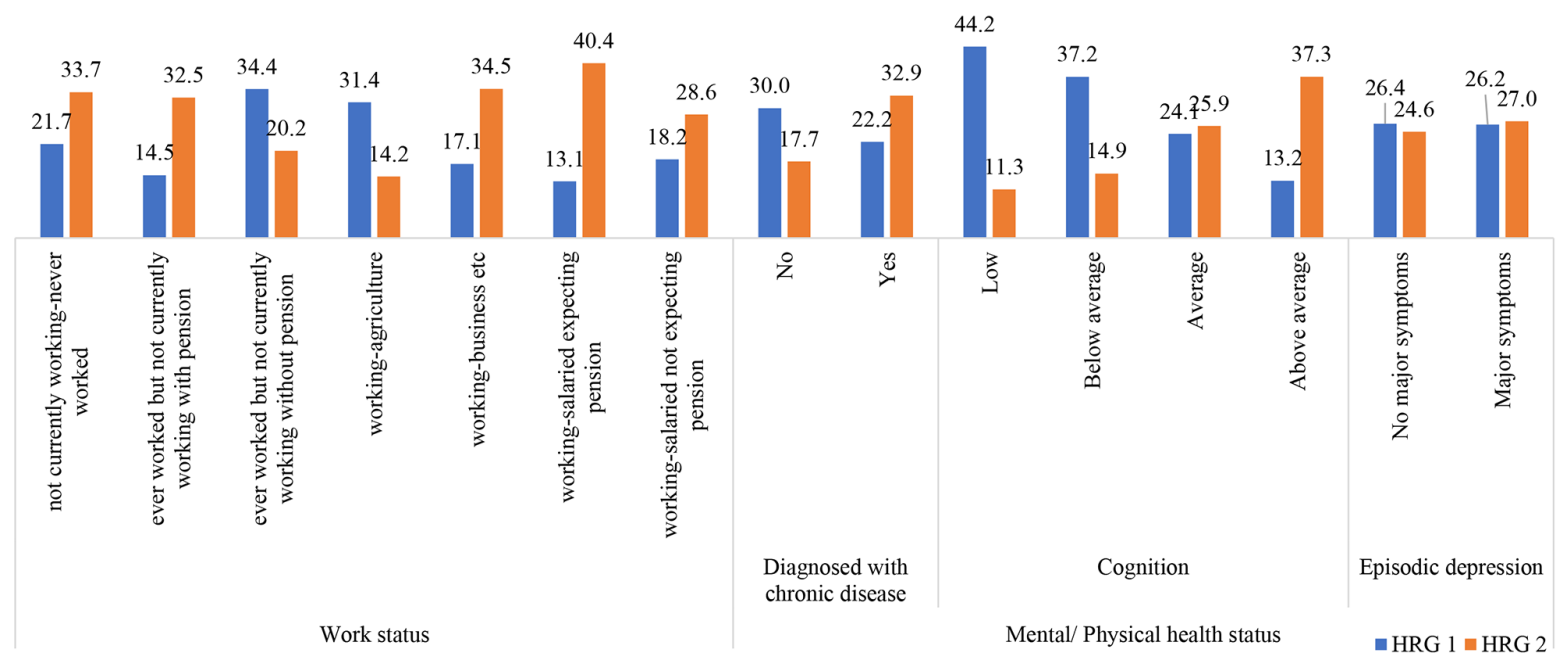
HRG categories by work status, physical and mental health status for adult respondents (45 years and above) in LASI, Wave 1, 2017-19

Figure 2 highlights the percentage of respondents belonging to either of the HRG by work status and physical/ mental health status. Out of the respondents who ever worked but are not currently working or receiving any form of pension and those engaged in agricultural work a higher percentage belonged to HRG1 compared to HRG2. Around 22.2% respondents with some diagnosed chronic disease belonged to HRG1 whereas 32.9% belonged to HRG2. The percentage of respondents belonging to HRG1 declined with increase in composite cognition score from 44.2% in the lower CCS category to 13.2% in the higher CCS category whereas the trend was opposite for HRG2 with 11.3% in the lower CCS category to 37.3% in the higher CCS category.

Relative risk ratios estimated from the multinomial logit GSEM are presented in Table 2. There was almost 20% higher chances of males belonging to HRG1 and 70% higher chances of females belonging to HRG2. Our study also found respondents who were 60 years and above had 30 to 40% higher chances of belonging to HRG1. Rural residents had 40% higher chances of belonging to HRG1 whereas urban residents had 70% higher chances of belonging to HRG2. Respondents with higher education and belonging to households in higher MPCE quintiles had lower chances of belonging to HRG1 and higher chances of belonging to HRG2. It is noteworthy that living arrangement of adults does not play a significant role in predicting their risk of being in HRG1. However, adults who live with their spouse and children had a higher risk of being in HRG2 (21–27% higher risk compared to the adults living alone). The result confirms that the relative risk of belonging to HRG1 was significantly higher (20–40%) among those respondents who ever worked but are not currently working or receiving pension and those engaged in agricultural work. Referring to the relative risk ratios from Table 2, it is observed that the risk of being in HRG2 doubles if the respondent was diagnosed with some chronic physical/ mental disease during the last 12 months. The risk of being in HRG1 is significantly lesser (by 4%) with an increase in composite cognition score. However, the risk of being in HRG2 increases by 3% with the increase in composite cognition score. The risk of being in HRG1 increases by 10% if the respondent suffers from episodic depression. The chances of belonging to HRG1 increases with lower life satisfaction whereas the chances of belonging to HRG2 increases with increase in life satisfaction index.

**Table 2:** Relative Risk Ratios from Multinomial logit Generalized Structural Equation Model on MUST categories estimated from LASI, Wave 1, 2017-19

Control variales	Response variable: MUST	
	HRG 1	HRG 2
**Demographic characteristics**		
**Gender**		
Male (Reference)		
Female	0.78*** (0.74,0.83)	1.72*** (1.63,1.83)
**Age**		
45-50yrs (Reference)		
50-60yrs	1.05 (0.98,1.13)	1.02 (0.96,1.08)
60-70yrs	1.25*** (1.17,1.35)	0.84*** (0.78,0.90)
70yrs+	1.42*** (1.31,1.55)	0.57*** (0.52,0.62)
**Religion**		
Hindu (Reference)		
Islam	0.79*** (0.73,0.86)	1.29*** (1.20,1.38)
Christian	0.51*** (0.47,0.57)	1.03 (0.94,1.13)
Others	0.71*** (0.62,0.80)	1.63*** (1.48,1.79)
**Caste**		
Scheduled Caste (Reference)		
Scheduled Tribe	1.01 (0.93,1.09)	0.75*** (0.69,0.82)
Other Backward Class	0.89*** (0.83,0.94)	1.05 (0.98,1.12)
None of them	0.84*** (0.78,0.91)	1.26*** (1.18,1.35)
**Living arrangements**		
Living alone (Reference)		
Living with spouse and/or others	0.96 (0.84,1.09)	1.21** (1.06,1.40)
Living with spouse and children	0.93 (0.82,1.05)	1.26** (1.11,1.44)
Living with children and others	1.04 (0.91,1.18)	1.27** (1.11,1.46)
Living with others only	1.00 (0.85,1.18)	1.02 (0.86,1.21)
**Education**		
No/ Less than Primary (Reference)		
Primary Completed	0.86*** (0.80,0.93)	1.35*** (1.26,1.44)
Middle Completed	0.85** (0.78,0.93)	1.39*** (1.29,1.50)
Secondary School/Matriculation	0.67*** (0.60,0.75)	1.55*** (1.43,1.68)
Higher Secondary/Intermmediate	0.72*** (0.62,0.83)	1.56*** (1.40,1.73)
Higher education	0.49*** (0.42,0.58)	1.63*** (1.47,1.80)
**MPCE**		
Poorest (Reference)		
Poorer	1.00 (0.94,1.07)	1.12** (1.04,1.21)
Middle	0.70*** (0.65,0.75)	1.10** (1.03,1.19)
Richer	0.67*** (0.62,0.72)	1.28*** (1.19,1.38)
Richest	0.70*** (0.65,0.76)	1.50*** (1.40,1.62)
**Work status**		
not currently working-never worked (Reference)		
ever worked but not currently working with pension	0.94 (0.80,1.11)	0.90 (0.79,1.02)
ever worked but not currently working with pension	1.40*** (1.30,1.50)	0.91* (0.86,0.98)
working-agriculture	1.24*** (1.15,1.33)	0.56*** (0.52,0.61)
working-business etc.	0.95 (0.85,1.05)	1.07 (0.98,1.16)
working-salaried expecting pension	0.99 (0.81,1.22)	1.01 (0.88,1.17)
working-salaried not expecting pension	0.96 (0.87,1.06)	0.82*** (0.76,0.90)
**Diagnosed with chronic disease**		
No (Reference)		
Yes	0.90*** (0.86,0.95)	2.05*** (1.96,2.14)
**Composite Cognition Scores**	0.96*** (0.95,0.96)	1.03*** (1.03,1.04)
**Episodic depression**		
No major symptoms (Reference)		
Major symptoms	1.10* (1.01,1.21)	1.06 (0.98,1.15)
**Life satisfaction Index (continuous scores)**	0.97*** (0.96,0.98)	1.02*** (1.01,1.03)
**Constant**	2.87*** (2.27,3.63)	0.04*** (0.03,0.05)

Note: *** *p*-value < 0.001; ** *p*-value < 0.01; * *p*-value < 0.05, the 95% CI’s are within parentheses

Additionally, the multinomial logit GSEM also captured the relative risk ratios of the array of health variables for various work categories (work/ pension/ financial security) on physical and mental health status. It is observed that there is 20% more risk of lower cognition and 10% more risk of lower life satisfaction among those who are not currently working and have no access to pension. Risk of lower cognition and life satisfaction was observed to be higher among the respondents who are currently working but are not expecting to avail any pension post-retirement compared to their counterparts who are currently working and are expecting pension post-retirement.

In Figure A.2 and A.3 (in Appendix) multinomial logit GSEM shows the results for male and female respondents after controlling for the structural variables (work status and physical/mental health) only. There are a few observations that needs introspection. For males, the risk of being in HRG1 increased 74% for those who ever worked but are not currently working and have no access to any form of pension and 40% for those who are engaged in agriculture. The risk of being in HRG2 for males suffering from episodic depression was 16% higher than their counterparts who did not report that they were suffering from episodic depression. The risk of being in HRG2 increased by 30–40% if a male respondent was engaged in business or salaried job with expected pension. For females, the risk of being in HRG1 increased by 43 to 50% if they were not currently engaged in any economic activity and 21% if they were engaged in agricultural work. Females suffering from episodic depression had 18% higher risk of belonging to HRG1. Thus, findings from the GSEM also shed light on gender differential risk of being a member of the high risk groups under the MUST framework.

## Discussion

The paper unravels the pathways through which financial security and physical and mental health affects the risk of being malnourished in adulthood. The dynamics and determinants of HRG1 and HRG2 were significantly different. The novel finding of the study elucidates that adults who are not covered by pension and works in informal sector like agriculture have significantly higher chances of lower cognition, low life satisfaction and episodic depression, being diagnosed with some chronic physical/ mental disease and in turn present higher risk of belonging to HRG1 (under-nutrition) of the MUST indicator. The study also brings to light the gender-based difference in the pathways through which financial security and physical/ mental health connects to risk of being malnourished in adulthood. Respondents at the better off end of the socio-economic spectrum had higher chances of belonging to HRG2.

Demographic characteristics such as gender, age, place of residence had a significant association with the risk of being in malnourished. Older females were more susceptible to HRG2 whereas oldermales had higher chances of belonging to HRG1. Existing research on adult malnourishment has pointed out that the growing burden of obesity among Indian adults, especially women is a rising public health concern [32]. A discernible structural shift is evident, where individuals in the lower economic strata continue to grapple with undernourishment, while those belonging to higher socio-economic strata has transitioned into sedentary lifestyles, leading to improved dietary habits, but consequently experiencing obesity and overnutrition [33]. The argument posits that women in India with higher socio-economic status, residing in urban areas, enjoying enhanced dietary variety, and having access to improved sanitation facilities have experienced an improved overall quality of life. However, this improvement has also heightened the risk of obesity and overweight. [34]. The increase in economic status, greater purchasing power, disparities in dietary choices, and cultural norms regarding ideal body sizes are believed to have exerted substantial influences on the prevalence of obesity and overweight among women, operating through intricate mechanisms [35–37]. Double burden of malnourishment, with underprivileged classes of the society suffering from undernutrition and privileged classes of the society suffering from overnutrition/ obesity has been a point of public health concern in the country [38]. Education and monthly per capita expenditure plays a significant role in determining membership in the high risk groups. Macroeconomic development in the country alone cannot counter the problem of malnourishment. With lack of sustainable food security and gaping gaps in nutrition supply, domestic supply and macronutrient availability remains a challenge for India [39]. Existing individual level social gradient in the risk of adult malnourishment can be mitigated only by reducing inequality and integrating the social dynamics into nutritional policies [40, 41].

Present working status, availability of pension and financial security has a direct effect on risk of malnourishment among adults. Respondents who belonged to “ever worked but not currently working without pension” and “working in agriculture” sectors had 20 to 40% higher chances of belonging to HRG1 and 10 to 40% lower chances of belonging to HRG2. However, this was significantly different by gender. Females who are not currently engaged in any work but have worked sometime in their lives and those engaged in agriculture had 20 to 48% higher chances of being undernourished. However, for males, those who are not currently working and not covered under any pension scheme and those who are engaged in agriculture have a higher risk of undernourishment. In absence of financial security, especially in the informal and agricultural sector, and presence of wide wealth-based inequalities in the country’s social welfare economics plays a significant role in the designing and execution of various policies. A significant portion of the India population, 42.6%, are still engaged in the agricultural sector in 2019 [42]. Most of the policies are pro-poor that cater to the poor, underprivileged and financially insecure population of the country. These policies are devised to provide safety nets to individuals and families that are underprivileged such as subsidized food supply through the Public Distribution System (PDS). PDS is one of the largest welfare programs aimed to provide food at a subsidized rate to poor households and has been documented to be effective in combating malnutrition [43]. However, many states of India do not have a universal PDS and often provides benefits to households below poverty line (BPL) and misses out resource poor households that do not qualify as a BPL household. The quality of the food grains provided by PDS, exclusion of resource poor households needs inspection to improve effectiveness of such welfare schemes [44–46]. Health shocks and costs of treatment remain a threat to poor households, especially households where majority of the members are engaged in the informal and agricultural sector [47].

Work status and availability or expectation of pension were significantly associated with cognition, episodic depression, life satisfaction and diagnosed chronic disease. Respondents who are not currently working but receiving pension, owns business, working with an expectation of receiving pension post-retirement had significantly higher cognition and life satisfaction compared to respondents who are not currently engaged in any work and do not receive any pension and those who are working but are not expecting any pension. This indicates that financial security is significantly associated with cognitive and mental health which indirectly determines the chances of belonging to high risk groups of malnourishment in adulthood. Previous research work shows that there is statistical significance to the comment “poverty impedes cognitive function”. A study published in science conducted a behavioral experiment and found that financial stress among the poor makes them act in less capable ways. The study concluded that financial constraints and related stress often has a significant negative impact on life satisfaction and cognitive function of individuals [48]. Financial worries and poverty often causes cognitive impairment which can increase the risk of malnourishment, especially undernourishment among the poor [49].

Our study found direct effects of low cognition and poor physical and mental health on risk of malnourishment of adults. The respondents who had been diagnosed with chronic diseases had significantly higher chances of belonging to HRG2. Respondents with higher cognition had 10 to 50% lower chances of belonging to HRG1 and 30 to 90% higher chances of belonging to HRG2. Mental and cognitive health issues is a silent killer and often remain undiagnosed for a long time. However, for older adults this problem is even more grave as a large percentage of older adults suffer from mental health issues such as anxiety and depression [11, 50]. However, there has been very limited study on cognition, mental health and their effects on the risk of malnourishment among adults and elderly, especially in a developing country. This study is one of the first ones to explore this linkage using a GSEM framework. The results from the study indicates inclusion of cognitive and mental health in nutrition policy frameworks for adults is imperative to attain the Sustainable Development Goal 2 of “zero hunger”.

## Conclusion

The findings of the study propose three primary arguments. Firstly, the strategies for addressing undernutrition and over-nutrition/obesity among adults should be distinct from one another due to the differing predictors associated with each condition. Secondly, it is noteworthy that chronic diseases and mental health disorders exert a considerable influence in predisposing undernutrition among adults employed within the informal sector, particularly those lacking any form of financial security, such as post-retirement pension benefits. The pathways by which mental health, physical health, and financial insecurity impact nutritional status among men and women differ, necessitating additional qualitative data to strengthen policy considerations. Thirdly, it is important to underscore that socio-economic advantages and financial stability are fostering a multitude of lifestyle habits that are elevating the risk of overnutrition and obesity within the adult demographic in India. The senior citizen population in the country has been projected to grow to 22.74 crore (14.9%) by 2036 calling for an urgent need to introspect on various adult and geriatric issues that can pose a threat to the public health sector. Malnourishment in adulthood can come with an array of comorbidities. Schemes such as Poshan Abhiyaan for Senior Citizens can be helpful in protecting senior citizens against financial insecurity and severe malnourishment [51]. Along with it psychosomatic disorders are an emerging public health issue. Understanding the linkage between adult malnutrition and health can shed light on policy and programme revisions that are essential for developing age-friendly infrastructure and policy planning in India. As India approaches the second demographic dividend, it is the need of the hour to dive deep into the relationship between mental and physical health to frame more inclusive policies. The study highlights the pathway in which financial insecurities and mental stress due to financial insecurity, retirement might affect adult malnourishment. This will provide rich insights on management, resource planning, and reformative measures that can be taken in establishing geriatric care in India which is looking forward to a speedy rise in the older population.

### Electronic supplementary material

Below is the link to the electronic supplementary material.

Supplementary Material 1

## Data Availability

The Longitudinal Ageing Study in India (LASI- Wave I), 2017-19 is a nationally representative survey of 72,250 older adults age 45 and above across all states and union territories of India. The data from the survey is available for public use on request. We requested for the raw data by filling up a form available at https://iipsindia.ac.in/sites/default/files/LASI_DataRequestForm_0.pdf. Details about how to acquire the data is available at https://www.iipsindia.ac.in/content/LASI-data.
