# Supplementary material for: Financial stress, health and malnourishment among older adults in India
Source: BMC Geriatr. 2023 Dec 15;23:861. doi: 10.1186/s12877-023-04532-7 (PMC10724991; doi:10.1186/s12877-023-04532-7)
Supplement: Supplementary file 1 — Supplementary Material 1 [file 12877_2023_4532_MOESM1_ESM.docx]

**Appendix**

**Figure A.1. Conceptual framework**

**Household socio-economic conditions**

**Living arrangement**

**Demographic profile**

**Financial security**

**Mental/ Physical health**

**Risk of malnutrition:**

- *Underweight*
- *Overweight*
- *Metabolic risk (waist to hip ratio)*
- *Food insecurity*
- *Hospitalization*

**Figure A.2. Generalized Structural Equation Model with response variable as the HRG 1 and 2 of MUST and structural variables-mental, cognitive, physical health and work status/ pension availability, Male, LASI, Wave 1**

**Figure A.3. Generalized Structural Equation Model with response variable as the HRG 1 and 2 of MUST and structural variables-mental, cognitive, physical health and work status/ pension availability, Female, LASI, Wave 1**

**Note: Abbreviations used in the abridged GSEM (multinomial logit): demographic and socio-economic variables not controlled**

must- Malnutrition Universal Screening Tool

Work- type of work and availability of pension

1.work-ever worked but not currently working with pension

2.work- ever worked but not currently working without pension

3.work- working-agriculture

4.work- working-business etc

5.work- working-salaried expecting pension

6.work- working-salaried not expecting pension

ccs-composite cognitive score

mh- mental health score

ls- life satisfaction score

cd- chronic disease
